# Serum opacity factor rescues fertility among female Scarb1^−/−^ mice by reducing HDL-free cholesterol bioavailability

**DOI:** 10.1016/j.jlr.2022.100327

**Published:** 2022-12-31

**Authors:** Corina Rosales, Dedipya Yelamanchili, Baiba K. Gillard, Jing Liu, Antonio M. Gotto, Henry J. Pownall

**Affiliations:** 1 Center for Bioenergetics and the Department of Medicine, Houston Methodist Research Institute, Houston, TX, USA; 2 Weill Cornell Medicine, Department of Medicine, New York, NY, USA

**Keywords:** Infertility, high-density lipoproteins, serum opacity factor delivery, cholesterol transport, gene therapy, HDL receptor gene, plasma HDL cholesterol, ovary morphology, ovary-free cholesterol content, bacterial virulence factor, AAV, adeno-associated virus, CERM, cholesteryl ester-rich particle, FC, free cholesterol, HDL-FCBI, HDL-FC bioavailability, LF, lipid-free, PL, phospholipid, Scarb1, gene coding for mouse SR-B1, SEC, size-exclusion chromatography, SOF, serum opacity factor, SR-B1, Scavenger Receptor Class B, Type 1, TC, total cholesterol

## Abstract

Human female infertility, 20% of which is idiopathic, is a public health problem for which better diagnostics and therapeutics are needed. A novel cause of infertility emerged from studies of female mice deficient in the HDL receptor gene (*Scarb1*). These mice are infertile and have high plasma HDL cholesterol (C) concentrations, due to elevated HDL-free cholesterol (FC), which transfers from HDL to all tissues. Previous studies have indicated that oral delivery of probucol, an HDL-lowering drug, to female Scarb1^−/−^ mice reduces plasma HDL-C concentrations and rescues fertility. Additionally, serum opacity factor (SOF), a bacterial virulence factor, disrupts HDL structure, and bolus SOF injection into mice reduces plasma HDL-C concentrations. Here, we discovered that delivering SOF to female Scarb1^−/−^ mice with an adeno-associated virus (AAV_SOF_) induces constitutive SOF expression, reduces HDL-FC concentrations, and rescues fertility while normalizing ovary morphology. Although AAV_SOF_ did not alter ovary-FC content, the ovary-mol% FC correlated with plasma HDL-mol% FC in a fertility-dependent way. Therefore, reversing the abnormal plasma microenvironment of high plasma HDL-mol% FC in female Scarb1^-/-^ mice rescues fertility. These data provide the rationale to search for similar mechanistic links between HDL-mol% FC and infertility and the rescue of fertility in women by reducing plasma HDL-mol% FC.

## Graphical Abstract

**Figure:**
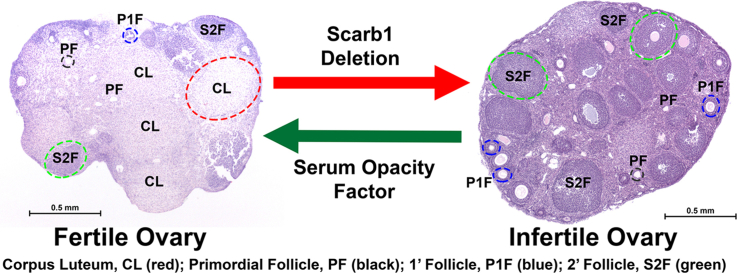

Human female infertility, some of which (∼20%) is idiopathic (1, 2), is a public health problem for which better diagnostics and therapeutics are needed. The influence of dysfunctional lipoproteins on female infertility is relatively unexplored, despite observations implicating them, especially HDL. In pregnant women, fetal development is associated with elevated HDL concentrations and the expression of placental HDL receptors that mediate HDL-free cholesterol (FC) transfer between the maternal circulation and the fetus (3). Early forms of HDL, that is, pre-β-HDL, have been implicated in the regulation of human placental lactogen expression during pregnancy (4). Lipoproteins transport lipids that are essential to fertility, including FC and steroid hormones, among tissues either directly or via their metabolites (5, 6). In humans, HDL, the only lipoprotein occurring in meaningful concentrations in the follicular fluid surrounding the developing oocyte in the ovary (7, 8, 9), may deliver lipids to the follicular cumulus cells and oocytes for membrane synthesis and multiple processes essential to oocyte maturation. The sizes and compositions of human serum- and follicular fluid-HDL are similar, the main difference being that relative to follicular fluid HDL, serum HDL is phosphatidylcholine-rich, deficient in lysophosphatidylcholine and acidic phospholipids (PLs), and containing a smaller fraction of HDL occurring as preβ_1_ HDL. (10) Given spontaneous, reversible FC transfer among lipid surfaces on a time scale of minutes (11), HDL is also a donor and acceptor of FC flux (12, 13, 14), which maintains FC homeostasis. Therefore, abnormalities in HDL metabolism that affect its structure, abundance, or function could compromise female fertility. Mice deficient in the HDL receptor, encoded by Scarb1, are a model of female infertility linked to dysfunctional HDL. Plasma HDL-FC concentrations among Scarb1^−/−^ mice are ∼7–10 times that of WT mice (15), which increases HDL-FC bioavailability (HDL-FCBI) expressed as the product of HDL particle number (HDL-P) and HDL-mol% FC (100 × moles_FC_/[moles_FC_ + moles_PL_]) (16, 17). Thus, HDL-FCBI = HDL-P x HDL-mol% FC. Notably, in this context of high HDL-FCBI, female Scarb1^-/-^ mice are infertile (18).

HDL is unstable because it resides in a kinetic trap from which it escapes in response to thermal and chaotropic perturbations, which typically displace APOA1 from the HDL surface and leave an APOA1-poor remnant (19, 20). All major HDL-modifying activities—esterification, (21) lipid transfer (22, 23, 24), lipolysis (22), SR-B1-mediated selective uptake (25), and disruption by bacterial serum opacity factor (SOF)—also alter HDL structure. (26) The effect of SOF is profound—in vitro, SOF quantitatively converts HDL to three products (supplemental Fig. S1): a small remnant called neo HDL, a large cholesteryl ester-rich particle (CERM) containing APOE plus the neutral lipids of >100,000 HDL particles, and lipid-free (LF) APOA1, the major HDL protein (26, 27, 28). Injecting SOF into mice diverts HDL cholesterol as CERM to APOE-mediated uptake by the hepatic LDL receptor with a subsequent ∼40% reduction in plasma HDL concentrations (29). Given that daily treatment of female Scarb1^−/−^ mice with probucol, an HDL-lowering drug, reduces HDL concentrations and rescues fertility among these mice, we tested the hypothesis that constitutively expressing SOF via an adeno-associated virus (AAV_SOF_) will rescue fertility among female Scarb1^−/−^ mice by reducing HDL concentrations and HDL-FCBI.

## MATERIALS AND METHODS

### SOF expression and isolation

Recombinant SOF, an 80 kDa truncated protein containing full opacification activity, was expressed and isolated from a bacterial expression system as previously described (26, 28).

### In vitro SOF kinetics

The kinetics of the SOF reaction versus WT and Scarb1^-/-^ mouse HDL isolated by sequential flotation at d = 1.063 and 1.21 g/ml were compared according to the changes in turbidity that are induced by the formation of CERM, large light-scattering particles that underlie the opacification phenomenon (26). Briefly, SOF (1 μg/ml) and HDL (1 mg/ml) were preincubated at 37°C, mixed in a temperature-controlled cuvette with a stir-bar within the cell compartment of an Aviv Model ATF 107 spectrofluorimeter also at 37°C, and the light scattering (325 nm) followed over time. Light-scattering versus time data were analyzed using a two-parameter rising exponential fit in SigmaPlot (Systat Software, Inc.). Rate constants are expressed as mean ± SD.

### Size-exclusion chromatography

Size-exclusion chromatography (SEC) was performed on an AKTA FPLC liquid chromatograph (GE-HealthCare, Inc.) equipped with two Superose HR6 size-exclusion columns. Column effluent was monitored by absorbance at 280 nm.

### AAV_SOF_ development and production

A pUCIDT-AMP plasmid encoding the SOF gene flanked by EcoRI and HindIII sites was synthesized by Integrative DNA Technologies. SOF DNA was isolated by restriction enzyme digestion and cloned into a pAAV-TBG-mcs plasmid. The pAAV-TBG-mcs-SOF plasmid was sequenced to confirm proper gene integration and orientation, amplified, and submitted to the University of Pennsylvania (UPENN) Vector Lab for virus production. Control pAAV-TBG-mcs-GFP plasmid was also purchased from UPENN. Transfection of Huh7 hepatocytes with pAAV-TBG-mcs-GFP confirmed good transcription efficiency, while transfection of pAAV-TBG-mcs-SOF confirmed expression and secretion of SOF protein.

### Mice

All mouse strains originated from The Jackson Laboratory (Bar Harbor, Maine). WT C57BL/6 mice, which exhibit good reproductive performance—a litter size of 6 ± 0.2 and a sterility rate of 8%, were used as controls. (30) Heterozygous Scarb1 (Scarb1^+/−^) breeders were used to derive homozygous Scarb1 knockout (Scarb1^−/−^) mice. Female Scarb1^−/−^ mice receiving AAV_SOF_ and nontreated male Scarb1^−/−^ mice were mated to generate Scarb1^−/−^ progeny. Mice were periodically genotyped to confirm genetic fidelity; expression of the targeted and WT Scarb1 alleles was confirmed by PCR amplification of DNA extracted from ear punches (primers 5′-GAT-GGG-ACA-TGG-GAC-ACG-AAG-CCA-TTCT-3′ and 5′-TCT-GTC-TCC-GTC-TCC-TTC-AGG-TCC-TGA-3′). Mice were maintained on a sterilized normal laboratory diet (Envigo). To optimize viral load, SOF-expressing and control mice were injected with AAV_SOF_ and AAV_GFP_, respectively, intraperitoneally at the rates of (0.3–2) × 10^11^ genome copies/mouse. AAV_SOF_ produced marked reduction in plasma total cholesterol (TC) and HDL-C levels.

### Fertility studies

During preliminary studies of AAV_SOF_ development, we observed that some of the female Scarb1^−/−^ mice produced pups. This observation provoked a systematic, controlled study which was performed in triplicate in groups of eight-week-old female Scarb1^-/-^ mice assigned to one of two treatments during which they received a normal laboratory diet (Harlan). One group (n_total_ 27, subgroups of 9 each) received probucol, which was added to their diet (0.5%, wt/wt). The other group (n_total_ 32, subgroups of 12, 11, and 9 each) received AAV_SOF_ (1.15 × 10^11^ genome copies). In addition, a small group of female mice received AAV_GFP_ to confirm that the AAV alone did not impact fertility. All groups were paired with male Scarb1^−/−^ mice for mating at eight weeks of age, that is, immediately following initiation of treatment with probucol or AAV_SOF_. The mice were followed for five months, during which they were maintained in a continuous monogamous breeding scheme in which the offspring were weaned between 21 and 28 days. Our metrics for fertility were number of days to first litter, litter size, percent fertile females, and survival expressed as the percent of litters surviving until weaning.

### Plasma and tissue FC analyses

Mice were sacrificed by CO_2_ inhalation, and plasma was collected by heart puncture followed by organ collection. Whole plasma and tissue lipids were determined using enzyme-based assays for FC, TC, PL, and triglyceride (Fujifilm Wako Diagnostics Inc.). Cholesteryl ester (CE) concentrations, determined as μg CE, were calculated as (μg TC – μg FC) × 1.6. Protein was determined by the DC Protein Assay (Bio-Rad, Inc.). The plasma, HDL, and ovary lipid analyses for WT and Scarb1^−/−^ have been previously reported (17) and are included here in Figs. 5 and 6 for comparison to the data for the AAV_SOF_–treated mice. Histology slides were prepared by the Pathology Core in the Department of Comparative Medicine Program at Houston Methodist Research Institute. Ovaries were fixed in 4% paraformaldehyde and embedded in paraffin blocks. Tissue blocks were cut into seven-μm serial sections by a microtome, mounted onto slides, and stained with hematoxylin/eosin for analysis.

### Statistical analysis

Data are mean ± SD or SEM, as indicated in the figure legends. Group means were compared by Student’s *t* test in Prism 9.2 or Microsoft Excel (Office 16). For comparisons between more than two groups, one way ANOVA for all groups was followed by Tukey comparison of mean values when the ANOVA p was significant, that is, *P*<0.05 (Prism 9.2).

### Study approval

All animal studies were approved by the institutional animal use and care committee.

## RESULTS

### WT and Scarb1^−/−^ mouse HDL is SOF-reactive

**Fig. 1:**
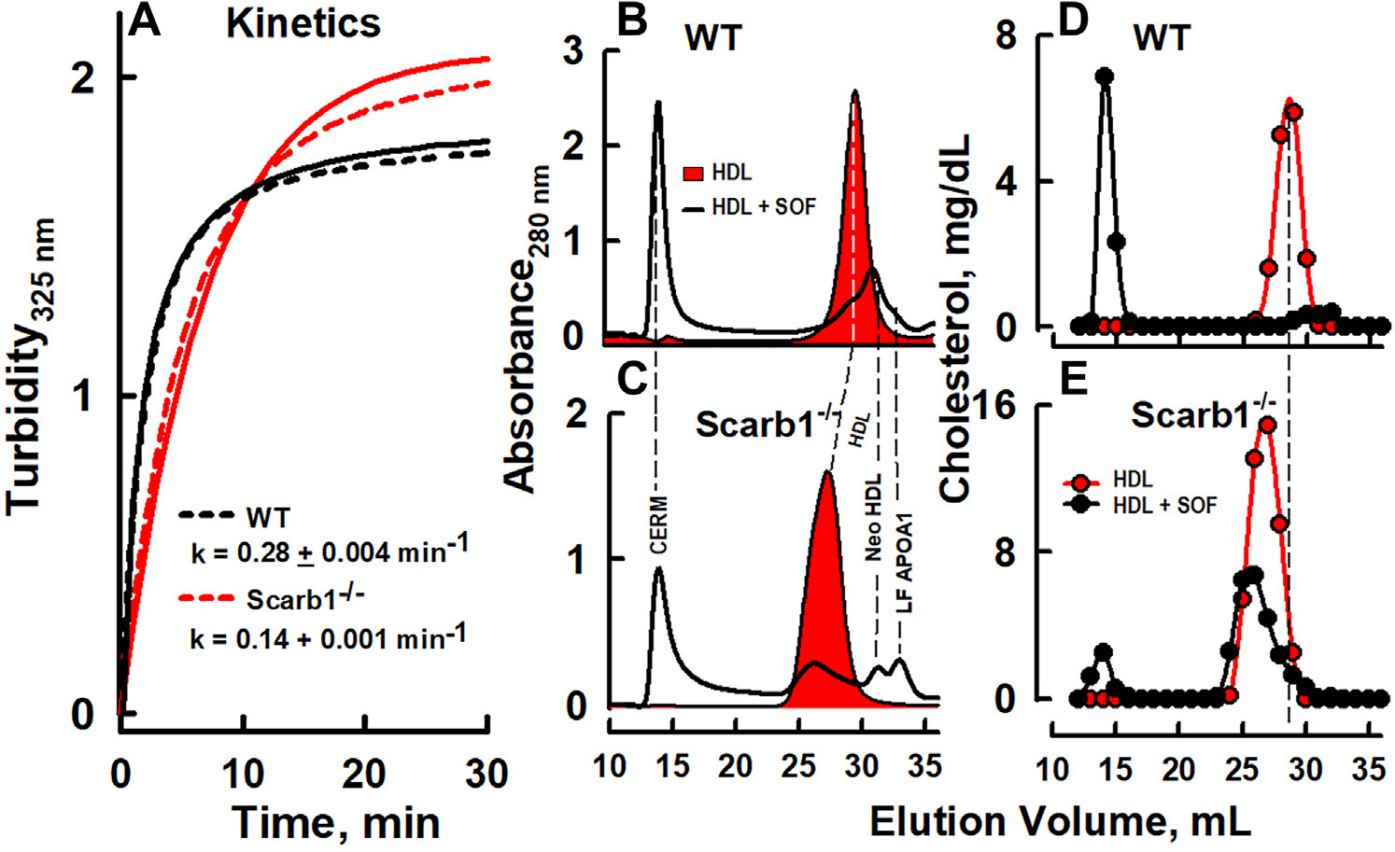
Serum opacity factor reaction. A: Kinetics of reaction versus HDL from WT and Scarb1^−/−^ mice as labeled. Dashed and solid lines are the experimental data and the data fitted lines, respectively, to a rising exponential fit. HDL (1 mg/ml) and SOF (1 μg/ml) were mixed at 37°C, and opacification was monitored by right-angle light scattering. B–E: SEC analysis of aliquots of the SOF reaction products versus HDL that were collected after completion of the kinetic studies. SOF incubated with HDL from WT (B, D) and Scarb1^−/−^ (C, E) mice. Profiles are expressed as protein absorbance (280 nm, B, C) and cholesterol concentration (D, E). The profiles with red fill are the protein absorbance (280 nm) profiles of HDL before treatment with SOF. SOF, serum opacity factor; SEC, size-exclusion chromatography.

We compared the in vitro effects of SOF versus HDL from WT and Scarb1^-/-^ mice by kinetic turbidimetry (Fig. 1A), by analyzing the reaction products by SEC, in which the eluent was monitored by absorbance (280 nm), which reflects the elution of protein (HDL, neo HDL, and LF APOA1) and light scattering for the CERM (elution volume = ∼15 ml; Fig. 1B, C), and by chemical analysis of the collected fractions (1 ml) for cholesterol (Fig. 1D, E). There were differences. First, the rate of reaction of SOF against HDL from Scarb1^−/−^ mice is ∼50% slower than that against WT HDL (Fig. 1A). Second, confirming previous reports (31, 32), HDL from Scarb1^−/−^ mice elutes earlier than that from WT mice (red-filled curves in Fig. 1B, C). In both groups, SOF catalyzed the formation of all three expected SOF products: CERM, neo HDL, and LF APOA1 (Fig. 1B, C). The SOF reaction versus HDL from WT mice was quantitative. In contrast, some HDL from Scarb1^−/−^ mice was unreactive even after a longer incubation of >3 h (data not shown). According to the cholesterol distribution, SOF catalyzed the transfer of ∼90% of WT mouse HDL-C to CERM (Fig. 1D), but only ∼15% of the HDL from Scarb1^−/−^ mice was converted to CERM by 30 min (Fig. 1E).

### AAV_SOF_ induces constitutive SOF activity in mice

**Fig. 2:**
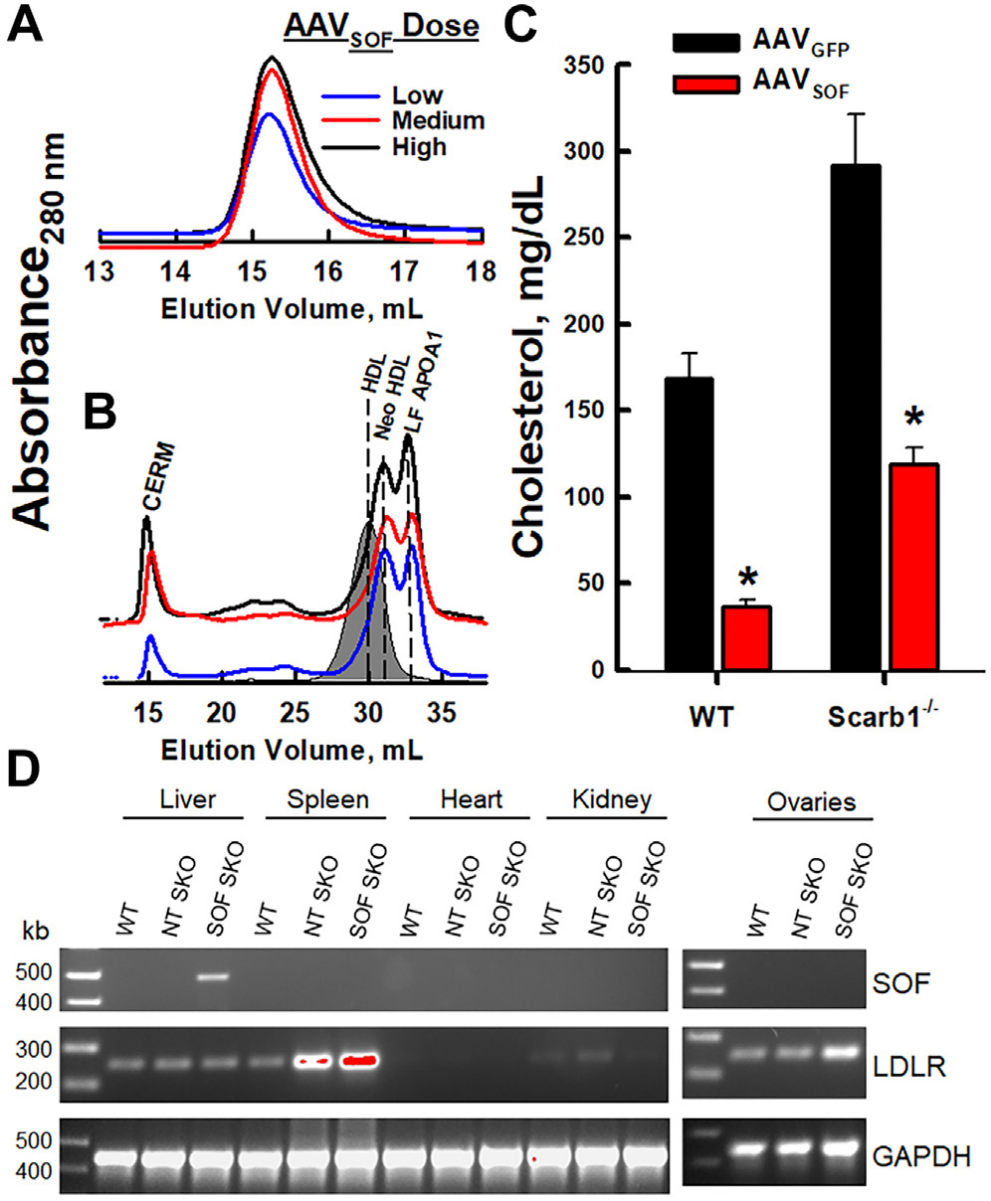
SEC of (A) mouse plasma at low (L—), medium (M—), and high (H—) AAV_SOF_ doses. B: SEC of 250 μl HDL (1 mg/ml) after incubation with plasma (5 μl) from mice receiving L-, M-, and H-dose AAV_*SOF*_. Gray-fill, HDL. C: Plasma total cholesterol 14 days after M-AAV_*SOF*_ dosing. ∗*P* < 0.05. D: PCR analysis of multiple tissues using primers for SOF, LDLR, and GAPDH as labeled for WT mice, Scarb1^-/-^ mice not treated with AAV_SOF_ (NT SKO), and Scarb1^-/-^ mice treated with AAV_SOF_ (SOF SKO). SEC, size-exclusion chromatography; SOF, serum opacity factor.

We conducted two tests of in vivo SOF activity following AAV_SOF_ injection into mice. First, we injected low, medium, and high AAV_*SOF*_ doses ([0.3, 1.15, and 2] × 10^11^ genome copies, respectively) into WT mice. One week postinjection, plasma was collected, and SOF activity was measured as CERM formation and analyzed by SEC. These data revealed a dose-dependent increase in CERM formation, thereby confirming that the mice expressed active SOF, which entered the plasma compartment and interacted with HDL to form CERM (Fig. 2A; A_280 nm_ peaks for neo HDL and LF APOA1 are obscured by LF plasma proteins). Given that the medium and high AAV_SOF_ doses gave similar responses, we conducted a second test in which we incubated plasma (5 μl) from the same mice with human HDL (250 μl, 1 mg/ml; 24 h, 37°C) and analyzed the reaction by SEC. Our data revealed a robust, quantitative conversion of HDL into the expected SOF products: CERM, neo HDL, and LF APOA1 (Fig. 2B). Injecting AAV_SOF_ into Scarb1^−/−^ mice effected a similar redistribution of HDL-C to CERM. Cholesterol analysis of the plasma from WT and Scarb1^−/−^ mice revealed that AAV_SOF_ catalyzes a profound reduction in plasma cholesterol concentrations (Fig. 2C). We also measured the expression of LDLR and SOF in multiple tissue sites (Fig. 2D). These data reveal the highest LDLR expression in liver and spleen and that SOF is only expressed in liver of the mice receiving AAV_SOF_; SOF mRNA was not observed in ovaries. Finally, the AAV_SOF_ modifies the distribution of APOA1 and APOE. Supplemental Fig. S2 shows the SEC profiles of plasma from WT, Scarb1^−/−^, and (Scarb1^−/−^ + AAV_SOF_) mice along with immunoblots for APOA1 and APOE according to fraction number. The effects of AAV_SOF_ delivery were similar to those we observed previously, (29) that is, following bolus SOF injection, APOE appears in the void volume where the CERM elutes and in the larger HDL subfractions from both Scarb1^−/−^ and (Scarb1^−/−^ + AAV_SOF_) mice. As expected, the only APOA1-positive band observed at Fraction 21, where neo HDL elutes, was from plasma of (Scarb1^−/−^ + AAV_SOF_) mice. Thus, the plasma of mice receiving AAV_SOF_ contains catalytically active concentrations of SOF that decrease plasma cholesterol.

### AAV_SOF_ rescues fertility and restores ovarian morphology among Scarb1^−/−^ mice

**Fig. 3:**
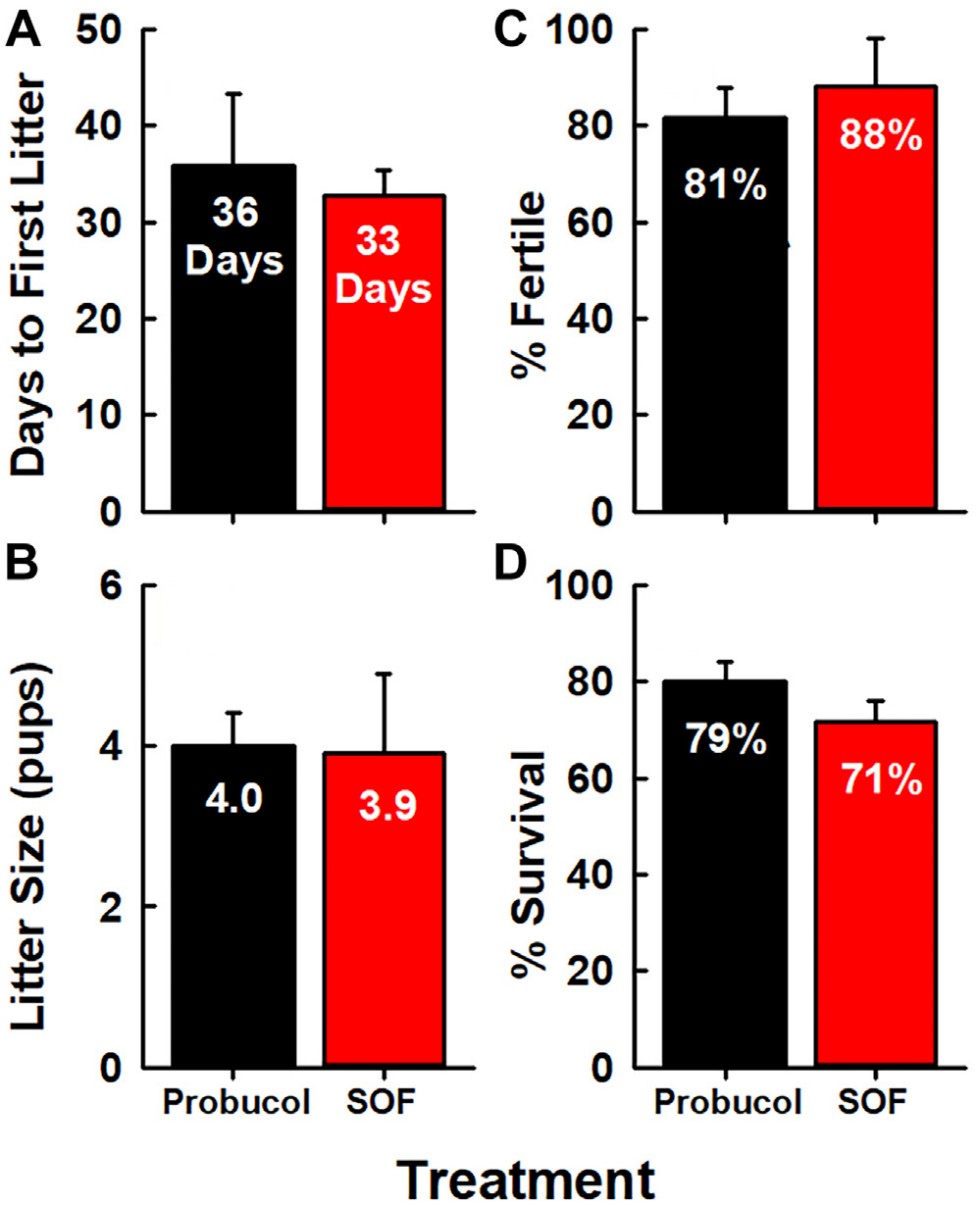
Fertility among female Scarb1^-/-^ mice receiving probucol or AAV_SOF_. Four markers of fertility were measured in three separate studies in which >10 mice per treatment were followed according to (A) the number of days until the first litter following treatment, (B) number of pups per litter, (C) percent of fertile females, and (D) percent surviving pups. Data are mean ± SD. Other details are in the Materials and methods section.

**Fig. 4:**
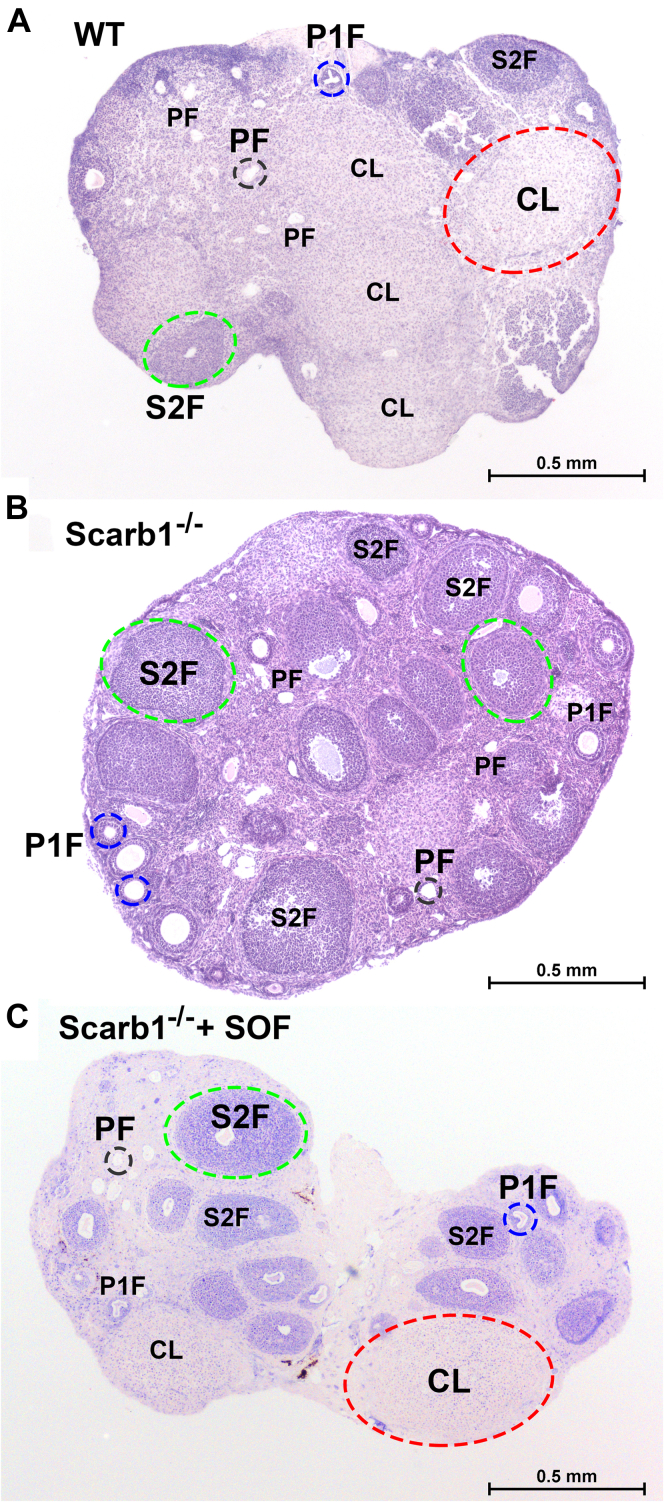
AAV_SOF_ normalizes ovary morphology. Panels show the representative histology of ovaries from (A) WT mice, (B) Scarb1^−/−^ mice, (C) Scarb1^−/−^ receiving AAV_SOF_. Primordial follicle, PF (black); primary follicle, P1F (blue); secondary follicle, S2F (green); corpus luteum, CL (red). Notably, the corpus luteum, which is not formed in ovaries from Scarb1^−/−^ mice, is restored by AAV_SOF_. The ages of the mice studied ranged from 12 to 20 weeks and there was no meaningful within-group difference among the ovaries.

We compared the fertility of female Scarb1^−/−^ mice receiving oral probucol with those given a single dose of AAV_SOF_. According to multiple criteria—time to first litter, litter size, and percent fertile mice—the rescue of fertility by AAV_SOF_ and probucol treatments (Fig. 3) was similar. Moreover, there was no difference in pup survival. The ovaries of mice in all three groups were collected and submitted for histological analysis. The effects of Scarb1 ablation on morphology were profound. The ovaries of WT mice contained the expected features associated with normal ovarian function. The major functional components could be clearly identified: primordial, primary, and secondary follicles and the conspicuously much larger corpus luteum (Fig. 4A). Qualitatively, these features were duplicated in the ovaries of Scarb1^−/−^ mice, but these mice also exhibited more oocytes/primordial follicles, fewer follicles undergoing maturation, and the total absence of corpora lutea (Fig. 4B), which is an essential feature of normal ovary morphology, function, and fertility. Additionally, compared to WT, ovaries from Scarb1^−/−^ mice appeared more fibrotic with less ovarian stroma, which may contribute to ovarian dysfunction. Similar studies of ovaries from Scarb1^-/-^ mice receiving AAV_SOF_ revealed that the rescue of fertility was associated with the recovery of normal ovary morphology, including the corpus luteum, a marker of successful ovulation (Fig. 4C).

### AAV_SOF_ alters plasma and HDL lipid compositions

**Fig. 5:**
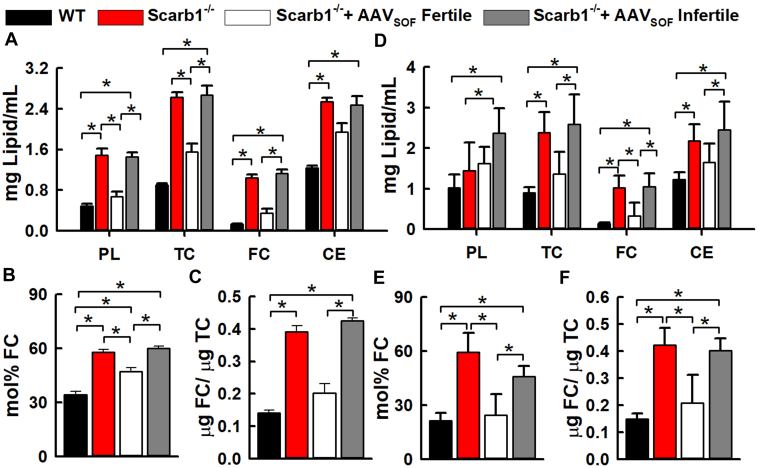
Lipid compositions of plasma (A–C) and HDL (D–F) from WT and Scarb1^-/-^ mice. As labeled, the mice were grouped as WT, Scarb1^−/−^ untreated, Scarb1^−/−^ treated with AAV_SOF_ and fertile, and Scarb1^-/-^ treated with AAV_SOF_ and infertile. (A) Plasma lipid compositions, (B) Plasma-mol% FC, (C) Plasma-FC/TC weight ratios, (D) HDL lipid compositions, (E) HDL-mol% FC, (F) HDL-FC/TC weight ratios. Mol% plasma FC = 100 × moles_FC_/(moles_FC_ + moles_PL_). The respective mean age ± SD and age ranges (weeks) were as follows: WT—20.4 ± 2.5 and 16–23; Scarb1^−/−^—19.5 ± 3.7 and 14–23; AAV_SOF_ Scarb1^−/−^ Fertile—39.5 ± 4.5 and 27–43; AAV_SOF_ Scarb1^−/−^ Infertile—42.8 ± 1.4 and 41–45. The respective number of mice per group were 12, 11, 12, and 13. FC, free cholesterol; TC, total cholesterol.

The lipid compositions, mol% FC, and FC/TC ratios of plasma and HDL from WT and Scarb1^−/−^ mice were compared. The latter included three groups: untreated Scarb1^-/-^ mice, fertile mice that had received AAV_SOF_, and infertile mice that had received AAV_SOF_. As previously reported, (17) Scarb1 deletion increased the plasma concentrations of all major lipids, the mol% FC, and the FC/TC ratio (Fig. 5A–C). Delivery of AAV_SOF_ to Scarb1^-/-^ mice reduced the plasma concentrations of all lipids, the mol% FC, and the FC/TC ratio among fertile mice, but not in AAV_SOF_-treated mice that remained infertile. The effects of AAV_SOF_ on HDL cholesterol concentration were similar. AAV_SOF_ decreased HDL-TC, FC, and cholesteryl ester (CE) to WT levels in fertile but not infertile Scarb1^-/-^ mice, as well as the mol% FC and the FC/TC ratio among fertile but not infertile mice (Fig. 5D–F).

### AAV_SOF_ alters ovary lipid compositions

**Fig. 6:**
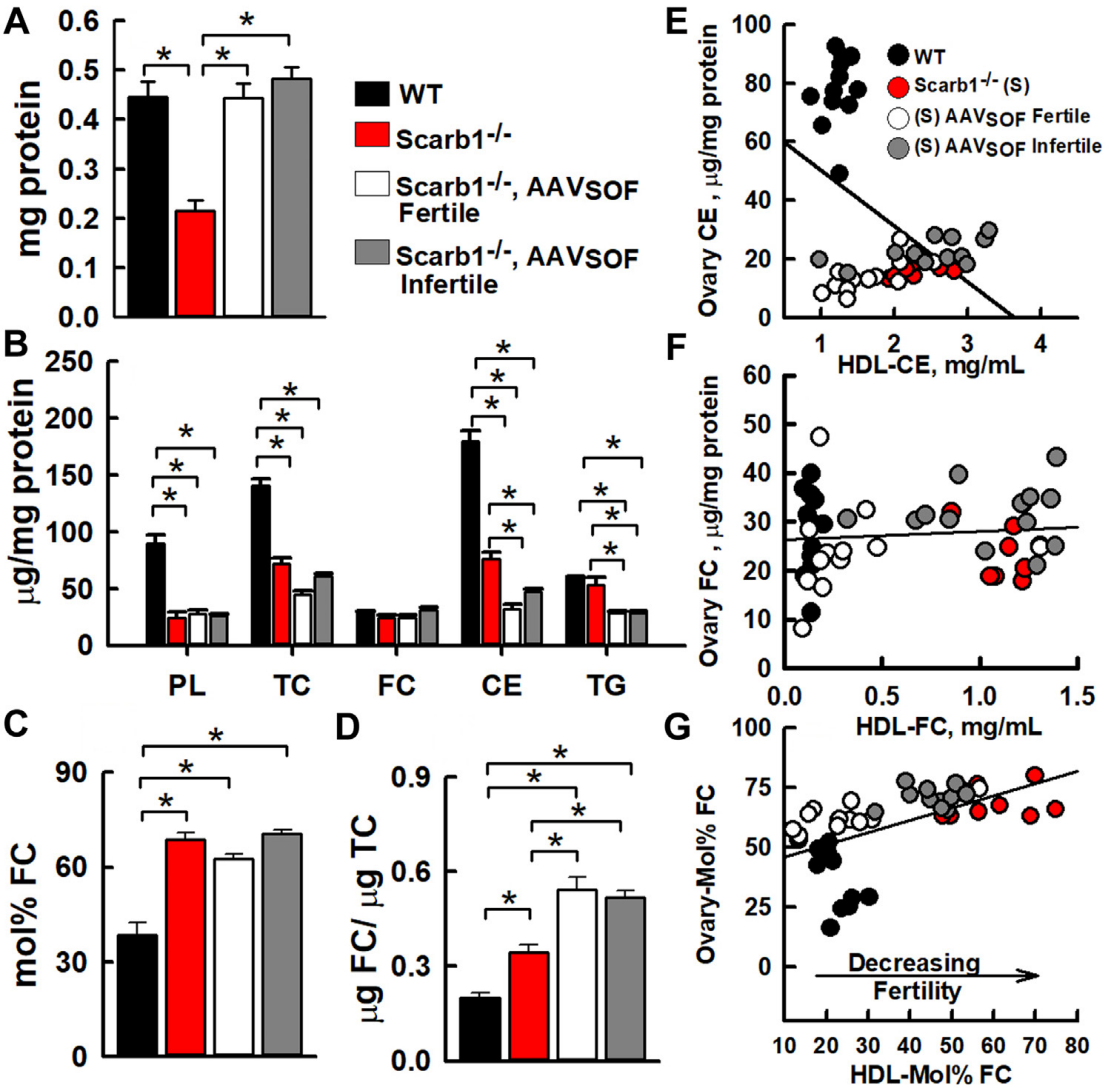
Lipid compositions of ovaries from WT and Scarb1^−/−^ mice. As labeled, the mice were grouped as WT, Scarb1^−/−^, Scarb1^−/−^ receiving AAV_SOF_ and fertile, and Scarb1^−/−^ receiving AAV_SOF_ and infertile. (A) Total protein per ovary, (B) ovary lipid compositions, (C) ovary mol% FC, (D) ovary FC/TC weight ratios. (E) Ovary CE content versus HDL-CE (m = −19 + 11; *r*^2^ = >0.05; *P* = 0.09), (F) Ovary FC content versus HDL-FC (m = 0.22 + 2.4; *r*^2^ = 0.33; *P* = 0.37), (G) Ovary mol% FC versus HDL mol% FC (m = 0.51 + 0.11; *r*^2^ = 0.33; *P* < 0.0001). Ovary mol%-FC = 100 × moles_FC_/(moles_FC_ + moles_PL_). Ages and number of mice per group are the same as in Figure 5 legend. FC, free cholesterol; TC, total cholesterol.

The ovaries of multiple mice were collected and analyzed. As a surrogate for ovary mass, we determined ovary protein and observed that ovary protein among Scarb1^−/−^ mice was less than half that of WT but restored to near WT values by AAV_SOF_ delivery to both fertile and infertile Scarb1^-/-^ mice (Fig. 6A). The effects of Scarb1 deletion and AAV_SOF_ treatment on ovary lipid compositions were distinct from the effects on plasma and HDL. Ovary PL was lower among Scarb1^−/−^ mice, an effect that was not reversed by AAV_SOF_ treatment. Ovary-FC was similar in all four groups, whereas ovary-CE for Scarb1^-/-^ mice was lower than that of WT. AAV_SOF_ further significantly reduced ovary-CE. The ovary-mol% FC was higher than that of WT among Scarb1^-/-^ mice and remained elevated in AAV_SOF_-treated mice (Fig. 6C). The ovary FC/TC ratio was significantly increased by Scarb1 ablation and further increased by AAV_SOF_ (Fig. 6D). We compared ovary versus HDL lipids among WT, Scarb1^−/−^, Scarb1^−/−^ + AAV_SOF_ (fertile), and Scarb1^−/−^ + AAV_SOF_ (infertile) mice (Fig. 6E–G). Comparison revealed a negative correlation between ovary-CE and HDL-CE; there was no correlation between ovary-FC and HDL-FC. In contrast, the ovary-mol% FC and HDL-mol% FC positively correlated. Thus, the restoration of fertility with AAV_SOF_ treatment correlates with reduction in the HDL- and ovary-mol% FC and not with ovary-FC or CE content.

## DISCUSSION

### HDL-FCBI

Within membranes and lipoproteins, PL and FC are confined to a common compartment in which the PL is the essential FC solvent. FCBI is determined by the relative amounts of FC and PL in these compartments and has been expressed as “accessible” (33, 34, 35), “active” FC (36, 37), and by the physico-chemical term, “fugacity” (37). We formulated a metric for HDL-FCBI calculated as the product of the number of HDL particles and the mol% FC within the particles (16), that is, mol% FC = 100 × X_FC_/(X_FC_ + X_PL_), where X_FC_ and X_PL_ are the number of moles of HDL-FC and PL, respectively. In a subsequent test, we reported that the high plasma mol% HDL-FC of Scarb1^-/-^ versus WT mice was associated with a high mol% FC in some but not all tissues (17). Notably, the ovary-mol% FC of Scarb1^−/−^ mice, which are infertile, was twice that of WT mice (17). Fertility among Scarb1^-/-^ mice is restored by inactivating the gene for the major HDL protein, APOA1, and by administering the HDL-lowering drug, probucol (18). Given that intravenous infusion of bacterial SOF transiently reduces plasma HDL-FC in mice (26), we tested the following hypotheses: a) infertility among female Scarb1^−/−^ mice is due to a high ovary mol%-FC provoked by a high HDL-mol% FC and b) constitutive SOF expression by AAV rescues infertility by reducing the ovary-mol% FC induced by a reduced HDL-mol% FC. Our hypotheses were only partly validated.

### SOF reaction versus Scarb1^−/−^ and WT HDL

The SOF reaction rate versus HDL from Scarb1^−/−^ mice is slower than that against WT HDL (Fig. 1A) and does not go to completion (Fig. 2), suggesting that WT and Scarb1^−/−^ HDL are structurally different and likely functionally different as well. The underlying cause of Scarb1^−/−^ HDL’s greater resistance than WT HDL to opacification may be differences in their structures. Scarb1^−/−^ HDL is larger than WT HDL: 12.5 and 10.2 nm, respectively. (15) However, SOF quantitatively converts various sizes of human HDL to the three expected products (26). The respective surface lipid compositions of WT and Scarb1^−/−^ HDL are also different: 17 and 58 mol% FC, respectively (15). As with apolipoproteins A1 and A2 (38, 39), the higher mol% FC in Scarb1^−/−^ versus WT HDL may make the former resistant to penetrance by SOF. Despite the resistance of some HDL from Scarb1^−/−^ mice to disruption by SOF in vitro (Fig. 2), fertility is still robustly rescued by AAV_SOF_, which induces constitutive SOF activity. The spontaneity of the SOF reaction supports the concept that both WT and Scarb1^−/−^ HDL reside in a kinetic trap (19) from which they can escape without energy input.

### Fertility

Female Scarb1^−/−^ mice are infertile due in part to excess oocyte FC, which induces premature activation and escape from MII arrest (40). We observed that ovaries from these mice have an abnormal morphology (Fig. 4). Ovaries of Scarb1^−/−^ mice were smaller, according to protein content, and the corpora lutea, markers of successful ovulation, were absent (Figs. 4 and 6A). In Scarb1^-/-^ versus WT mice, the less abundant ovarian stroma—the connective tissue that binds the ovarian structures, including the follicles, together—could contribute to infertility. Ovarian stromal cells are important in follicle development, hormone production and responsiveness, and ovulation, and the lower abundance of ovarian stromal cells could explain the absence of mature follicles in the Scarb1^-/-^ ovaries. The greater fibrosis in Scarb1^−/−^ ovaries, which are characterized by more fibroblasts and extracellular matrix, than in WT ovaries could also reduce ovarian function and fertility.

The effects of AAV_SOF_ and probucol on fertility were similar, including time to first litter, litter size, fertility rate, and pup survival (Fig. 3) (18). In addition, AAV_SOF_ normalized ovary morphology, including organ size and the appearance of corpora lutea (Figs. 4 and 6A). Scarb1 ablation reduces ovary PL and CE and increases ovary-mol% FC and FC/TC ratio. If these changes were mechanistically linked to infertility, one would expect one or more of them to approach WT values with the rescue of fertility; this was not observed (Fig. 6B–D). Ovary lipid compositions did not correlate with infertility and its rescue.

We then studied whether the lipid compositions of plasma, which comprise the ovary micro-environment, determine fertility. In mice, HDL is the dominant lipoprotein species (41), so we focused on comparing HDL lipids and ovary lipids (Figs. 5 and 6). Our study revealed no correlation between ovary-FC and HDL-FC concentration (Fig. 6F, *P* = 0.37). It is striking that the concentration of FC in ovaries was constant across all four groups tested (Fig. 6B), even though CE varied by six-fold between the WT and Scarb1^−/−^ groups, indicating the importance of maintenance of FC homeostasis in ovaries. Moreover, ovary-CE and HDL-CE concentration showed a weak inverse correlation (Fig. 6E, *P* = 0.09) and would not be expected to affect cells because, in mice, CE are metabolically silent. First, CEs are confined to the core of HDL, so their interactions with cells are blocked by the HDL-surface monolayer of PL and proteins. Second, mice do not express cholesteryl ester transfer protein, which moves CE to the APOB-containing lipoproteins for hepatic extraction. Lastly, because they lack SR-B1, the HDL receptor, ovarian CE uptake is limited in Scarb1^−/−^ mice. In contrast, ovary-mol% FC and HDL-mol% FC positively correlated (*P* = 0.0001). HDL bioavailability, measured as mol% FC, could contribute to infertility via interactions with ovaries but without the net transfer of FC, even though FC can spontaneously transfer through the aqueous phase between lipid surfaces without a carrier. (11) While the change in the mol% HDL-FC is large, the mechanism by which such a change in the microenvironment could induce such profound changes in ovary biology is not immediately clear.

The relevance of mol% FC in fertility is illustrated by studies of PDZK1^-/-^ mice (42), which have lower SR-B1 protein expression than WT mice in liver and intestine, but not in ovary, adrenal, and testis. Moreover, PDZK1^−/−^ mice are fertile despite plasma lipoprotein profiles that are similar to those of Scarb1^-/-^ mice. As with Scarb1^−/−^ mice, their plasma cholesterol concentration is nearly twice that of WT mice, and their HDL is larger and APOE-rich. However, their plasma mol% FC was lower (26 mol%) than that of both WT (34 mol%) and Scarb1^−/−^ mice (57 mol%). Given that nearly all plasma cholesterol in mice is carried by HDL, the differences in the plasma mol% FC are likely due to underlying differences in HDL mol% FC. These data showing that a lower HDL-mol% FC among PDZK1^−/−^ versus Scarb1^−/−^ mice, despite otherwise similar plasma lipoprotein profiles, is associated with normal fertility provide independent support for the hypothesis that a plasma microenvironment of a high HDL-mol% FC contributes to infertility. Although excess HDL-FC does not increase the FC content of all tissues, (17) all tissues are in contact with a high concentration of HDL-FC, which may initialize a signal transduction pathway of unknown identity, which has been described as “a complex interaction between the ovary and the extraovarian environment (i. e., the hypothalamic-hypophyseal ovarian axis)”(18).

The rescue of fertility among Scarb1^−/−^ mice by probucol and AAV_SOF_ confirmed the finding that ovary SR-B1 expression is not needed for fertility (18). Probucol and AAV_SOF_ induce similar reductions in plasma lipid concentrations, but with different underlying effects. Probucol reduces concentrations of the large HDL particles formed in Scarb1^-/-^ mice (18). In contrast, AAV_SOF_ induces a greater reduction in the concentration of the small HDL particles. Thus, the ovary-toxic effects of high HDL concentrations are independent of HDL particle size. The mechanisms are also different. Probucol reduces plasma HDL concentrations, in part, by reducing HDL synthesis (43). SOF disrupts HDL structure (28) thereby diverting HDL-C to the hepatic low-density lipoprotein receptors via large APOE-containing particles, CERM, that contain nearly all HDL-C (26, 29). Although the cholesterol-lowering effects of probucol and AAV_SOF_ were similar, the former requires daily oral delivery, whereas a single AAV_SOF_ dose effected constitutive cholesterol lowering lasting more than 30 weeks and restored fertility among Scarb1^-/-^ mice.

Some human genetic alterations in *SCARB1* adversely impact cholesterol metabolism in the ovaries, thereby reducing fertility, and although some *SCARB1* single-nucleotide polymorphisms are associated with human female infertility, underlying mechanisms are not known (44, 45). The only human loss-of-function *SCARB1* mutation reported was a female with two children (46). Thus, the SCARB1-fertility axis likely involves contributions from other genes.

### Conclusions

Nearly all clinical laboratories determine TC, which comprises FC and CE, each of which has a distinct metabolic itinerary, so that connections between HDL-FC and various pathologies, including infertility, elude detection. Our findings provoke the hypothesis that very high plasma HDL-FC concentrations also underlie some forms of human female infertility. This hypothesis can be readily tested using routine lipid tests as described above to compare the HDL-FC concentrations of women visiting a clinic for in vitro fertilization with the HDL-FC concentration of control fertile women. Globally, nearly two million cycles of in vitro fertilization are performed annually (47) at a conservatively estimated cost of $10,000 per cycle. Thus, the option for an inexpensive medical solution is attractive even if it only benefits a small subset of women with HDL-associated infertility. Validation of our hypothesis linking high HDL-FC concentrations with infertility would provoke tests of HDL-FC-lowering therapies such as probucol or even SOF as fertility agents in infertile women with a high plasma HDL-FC.

### DATA AVAILABILITY

All data are contained within the article.

## Supplemental data

This article contains supplemental data.

## Conflict of interest

The authors declare that they have no conflicts of interest with the contents of this article.
