# Supplementary material for: Serum opacity factor rescues fertility among female Scarb1−/− mice by reducing HDL-free cholesterol bioavailability
Source: J Lipid Res. 2022 Dec 31;64(2):100327. doi: 10.1016/j.jlr.2022.100327 (PMC9932678; doi:10.1016/j.jlr.2022.100327)
Supplement: Supplemental figure 1 [file mmc1.pdf]

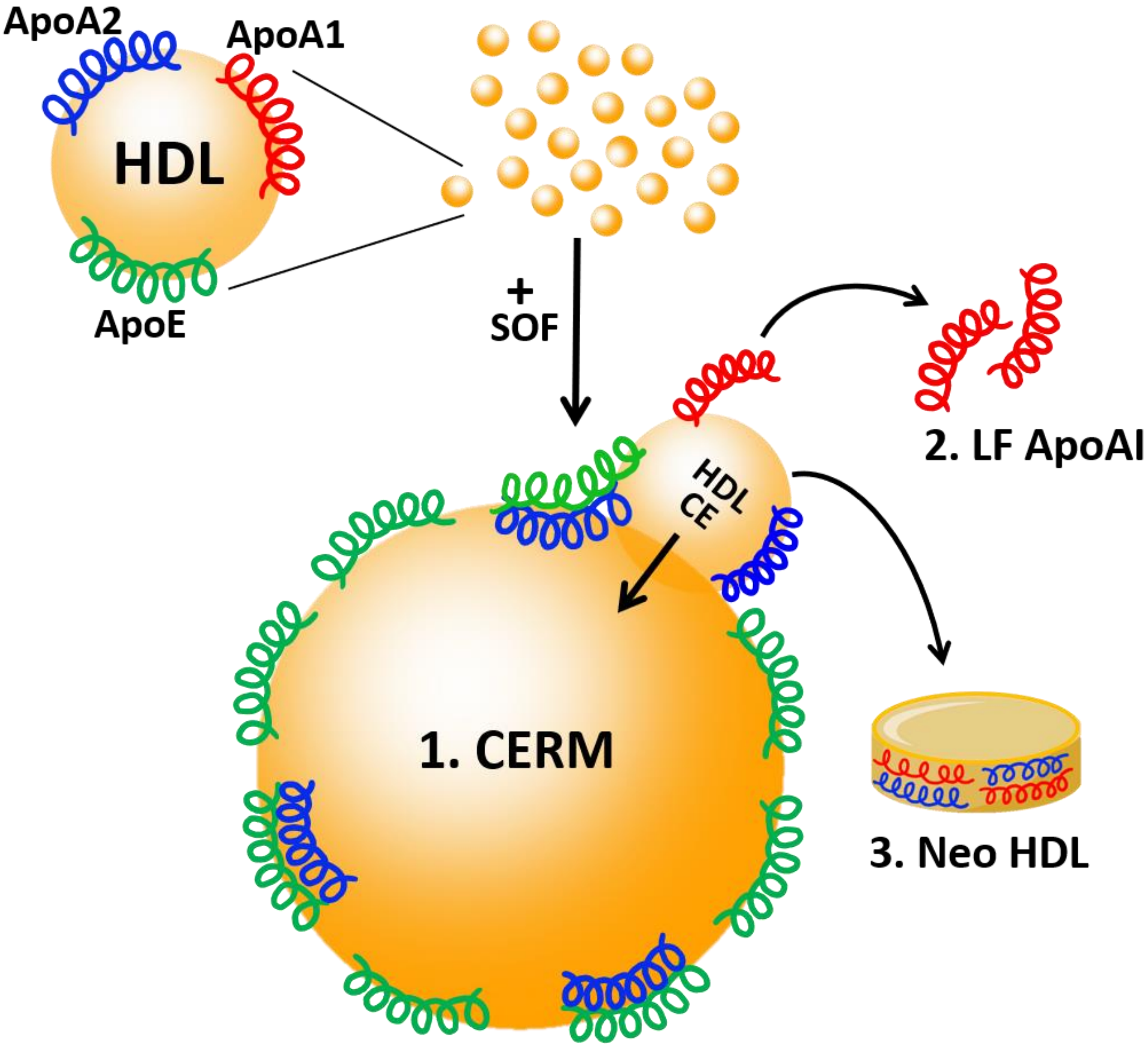

Supplementary Figure 1: Summary of the Reaction of SOF vs. HDL. SOF interacts with HDL resulting in the physical binding of two or more HDL particles causing cholesteryl ester lipid transfer to one main HDL host. The reaction gives rise to three distinct products: (1) a large cholesteryl ester-rich particle (CERM) that contains nearly all the neutral lipids of >100,000 HDL particles, (2) lipid-free (LF) APOA1, and (3) a small HDL remnant (neo HDL). The CERM contains APOE and its dimer with APOA2 as its sole proteins.
