# Supplementary material for: Serum opacity factor rescues fertility among female Scarb1−/− mice by reducing HDL-free cholesterol bioavailability
Source: J Lipid Res. 2022 Dec 31;64(2):100327. doi: 10.1016/j.jlr.2022.100327 (PMC9932678; doi:10.1016/j.jlr.2022.100327)
Supplement: Supplemental figure 2 [file mmc2.pdf]

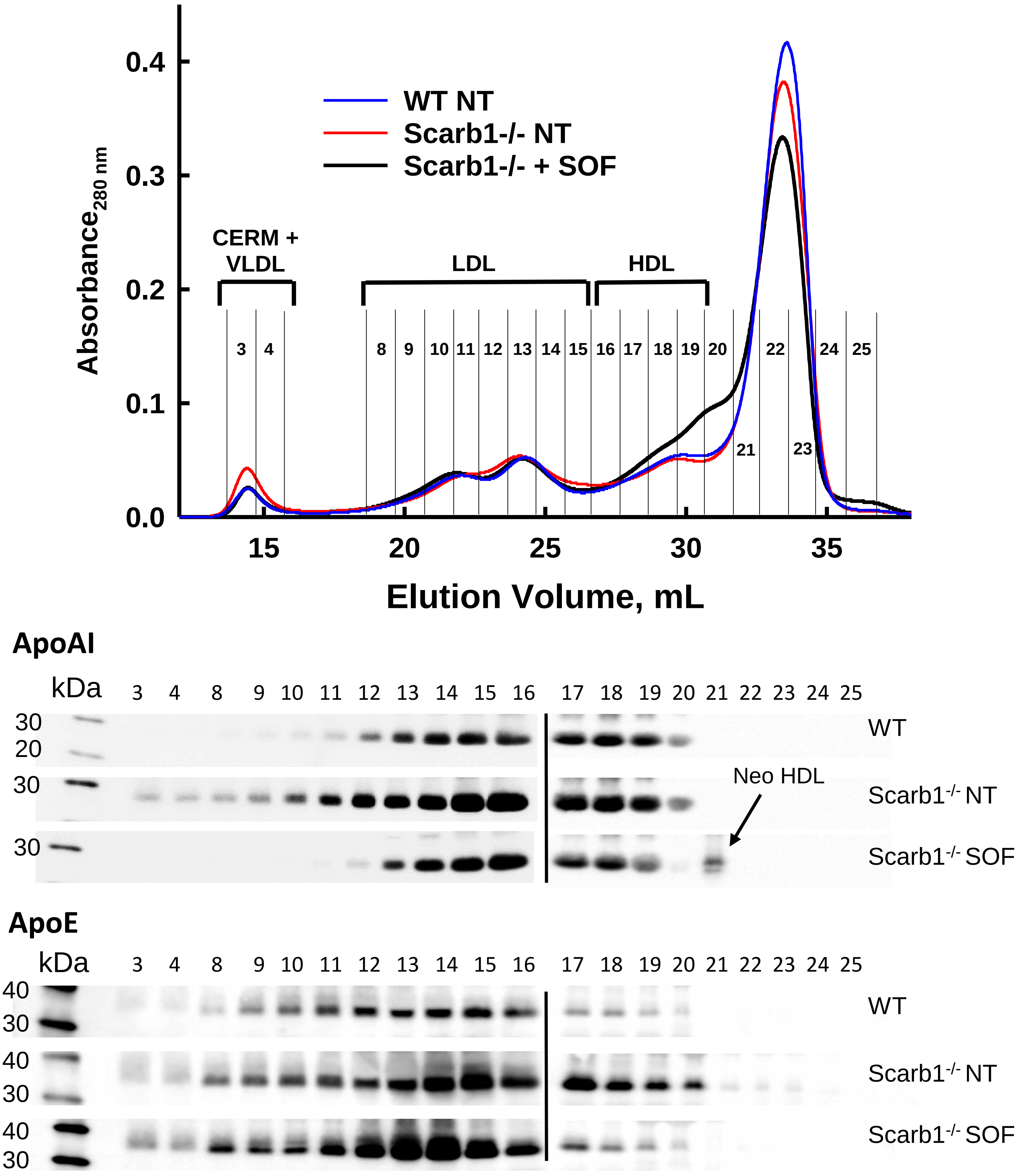

Supplementary Figure 2: SEC of Plasma from Mice according to Absorbance (280 nm) and Immunoblots of the Collected Fractions as Labeled. There were five mice per group—WT (17 weeks old), Scarb1-/- (21 weeks old), and Scarb1-/- + AAV_{SOF} (21 weeks old), which had received AAV_{SOF} for 3 weeks prior to sacrifice. SEC and immunoblot data are representative. Plasma loading was loading was 15 µL for lanes 3, 4, and 8-20 and 2 µL for lanes 21-25,
